# Long non-coding RNAs enable precise diagnosis and prediction of early relapse after nephrectomy in patients with renal cell carcinoma

**DOI:** 10.1007/s00432-023-04700-7

**Published:** 2023-03-29

**Authors:** Julia Bohosova, Katerina Kozelkova, Dagmar Al Tukmachi, Karolina Trachtova, Ondrej Naar, Michaela Ruckova, Eva Kolarikova, Michal Stanik, Alexandr Poprach, Ondrej Slaby

**Affiliations:** 1 Masaryk University, Central European Institute of Technology, Kamenice 753/5, 625 00 Brno, Czech Republic; 2 Masaryk Memorial Cancer Institute, Department of Comprehensive Cancer Care, Faculty of Medicine, Masaryk University, Zluty Kopec 543/7, 602 00 Brno, Czech Republic; 3 Faculty of Medicine, Department of Biology, Masaryk University, Kamenice 753/5, 625 00 Brno, Czech Republic

**Keywords:** Long non-coding RNA, Diagnosis, Prognosis, Biomarker, Early relapse, Next-generation sequencing

## Abstract

**Purpose:**

Renal cell carcinoma belongs among the deadliest malignancies despite great progress in therapy and accessibility of primary care. One of the main unmet medical needs remains the possibility of early diagnosis before the tumor dissemination and prediction of early relapse and disease progression after a successful nephrectomy. In our study, we aimed to identify novel diagnostic and prognostic biomarkers using next-generation sequencing on a novel cohort of RCC patients.

**Methods:**

Global expression profiles have been obtained using next-generation sequencing of paired tumor and non-tumor tissue of 48 RCC patients. Twenty candidate lncRNA have been selected for further validation on an independent cohort of paired tumor and non-tumor tissue of 198 RCC patients.

**Results:**

Sequencing data analysis showed significant dysregulation of more than 2800 lncRNAs. Out of 20 candidate lncRNAs selected for validation, we confirmed that 14 of them are statistically significantly dysregulated. In order to yield better discriminatory results, we combined several best performing lncRNAs into diagnostic and prognostic models. A diagnostic model consisting of AZGP1P1, CDKN2B-AS1, COL18A1, and RMST achieved AUC 0.9808, sensitivity 95.96%, and specificity 90.4%. The model for prediction of early relapse after nephrectomy consists of COLCA1, RMST, SNHG3, and ZNF667-AS1 and achieved AUC 0.9241 with sensitivity 93.75% and specificity 71.07%. Notably, no combination has outperformed COLCA1 alone. Lastly, a model for stage consists of ZNF667-AS1, PVT1, RMST, LINC00955, and TCL6 and achieves AUC 0.812, sensitivity 85.71%, and specificity 69.41%.

**Conclusion:**

In our work, we identified several lncRNAs as potential biomarkers and developed models for diagnosis and prognostication in relation to stage and early relapse after nephrectomy.

## Introduction

Despite the great efforts and progress in imaging techniques and therapeutical options in the last decades, renal cell carcinoma (RCC) still remains one of the deadliest urogenital cancers worldwide, being the ninth most common neoplasm in the United States and accounting for 2% of global cancer cases (Padala et al. 2020). The incidence of RCC rises especially in higher income regions where accessibility of primary health care and imaging techniques enables early identification of RCC cases (Capitanio et al. 2019). However, more than 50% of cases are discovered incidentally (Escudier et al. 2019); as only a small fraction of patients present with typical symptoms of RCC, and no systemic screening programe has been developed so far (Decastro and McKiernan 2008; Padala et al. 2020). And still, even the growing accessibility of imaging does not cover the diagnostic need, as the locally advanced disease is already present in a notable proportion of patients, with almost 17% harboring distant metastasis (Capitanio et al. 2019). Moreover, a significant portion of patients after successful nephrectomy experience relapse relatively early, up to 2 years after the surgery. Currently recognized prognostic models, namely stage, size, grade, and necrosis (SSIGN) score, the University of California Los Angeles Integrated Staging System (UISS), and molecular models such as ClearCode34 can provide only limited prognostic value. No preference is placed on any of them by official oncology guidelines (Escudier et al. 2019). Assessment of prognosis is currently based mainly on histological and clinical factors, and the pressing clinical need to predict early relapse after nephrectomy stays unmet as no reliable biomarker has been discovered and implemented yet.

Long non-coding RNAs are such potential biomarkers, as their expression is dysregulated in association with many human diseases not only as a secondary effect but also as a causative event (Chi et al. 2019). As potent regulators of gene expression on all levels from chromatin organization all the way to the protein folding and beyond, lncRNAs have a vital impact on cell functioning (Quinn and Chang 2016; Chi et al. 2019). Dysregulation of lncRNA levels affects other cellular components and contributes to development of pathologic conditions but can also serve as a unique snapshot of current situation in any given cell (Chandra Gupta and Nandan Tripathi 2017; Bohosova et al. 2021). In the recent decade, there was a plethora of publications focused on dysregulation of lncRNA in many human diseases, although, predominantly in cancer, including renal cell carcinoma (Chandra Gupta and Nandan Tripathi 2017; Bhan et al. 2017).

In a present study, we aimed to identify diagnostic and prognostic lncRNAs, specifically dysregulated in patients with early relapse after nephrectomy and thus provide potential new tool for diagnosis and prognostication of patients in higher risk of disease progression.

## Materials and methods

### Samples and patients

Patients diagnosed with renal cell carcinoma were enrolled during their treatments at the Masaryk Memorial Cancer Institute, Brno, the Czech Republic, between 2014 and 2018. Enrollment, sample, and clinical data management were handled according to the Declaration of Helsinki. Signed informed consent has been collected from all the patients before the study enrollment. The study design was approved by the Ethics Committee of the Masaryk Memorial Cancer Institute and Ethics Committee of Masaryk University. From all 259 patients, tumor renal parenchyma and adjacent non-tumor tissue have been taken during the nephrectomy. All tissue was first submerged in RNA-later and then stored at -80 °C until further processing. In Table 1 relevant clinical characteristics for the select cohorts of patients used in sequencing and validation part of the study are shown.

**Table 1:** Select clinical characteristics of the patient cohorts

Characteristic	Sequencing cohort	Validation cohort
Number of patients/samples	48/96	198/394
Sex		
Women	14	52
Men	34	145
Age at the time of the diagnosis		
Median (years)	64	64
Range (years)	36–83	31–87
RFS < 25 months early relapse	24	15
RFS 25–50 months	–	9
RFS > 50 months	24	159
No relapse, short follow-up*	–	15
Stage		
I	18	150
II	11	19
III	19	17
IV	0	11
Histology Clear cell Chromophobe Papillary (both types) Sarcomatoid Combination Other	37 4 2 1 4 0	163 10 15 2 5 2

*No relapse, but the patient wasn´t followed for at least 50 months due to death

Out of the 259 patients enrolled in the study, several (*N* = 13) patients had to be excluded due to extremely short overall survival after nephrectomy, and death unrelated to the disease relapse (up to ten months from surgery), or invalid clinical data. The remaining 246 patients were stratified into 2 independent cohorts for the sequencing and validation part of the study. The sequencing cohort of 48 patients consisted of two groups which were later compared with each other—early relapse and late-relapse, both groups contained 24 patients (together 96 samples, as we processed both the tumor and non-tumor tissue from each patient). Early-relapse patients were defined as relapses up to 25 months after nephrectomy (median 10,58 months, range 2.41–21.97). Late-relapse group consisted of patients without relapse events for the period of more than 50 months after the nephrectomy. For validation, we used all the remaining patients a divided them again into two groups—early and late-relapse patients. Early-relapse group consisted of all the remaining patients (*N* = 15) who experienced relapse before the 25-month mark. In the late-relapse group were all the remaining patients (*N* = 183) who relapsed after 25 months or had not relapsed at all during the 5-year follow-up.

### Isolation and quality control

Frozen tissue samples were thawed at the processing site and from each specimen, a 0.5 × 0.5 cm piece was cut and further processed. Total RNA enriched for small RNAs was extracted using mirVana™ miRNA isolation kit (Invitrogen, Waltham, MA, USA) with a minor alteration of the manufacturer’s protocol. Specimens were first homogenized using ceramic beads (Qiagen, Hilden, Germany) along with the Lysis/Binding buffer from the mirVana™ miRNA isolation kit (Invitrogen, Waltham, MA, USA). We then proceeded to the RNA extraction as suggested by the manufacturer. The concentration of the extracted RNA was measured using NanoDrop 2000 Spectrophotometer (Thermo Fisher Scientific, Waltham, MA, USA). Extracted RNA was stored at − 80 °C until further processing.

### Library preparation and transcriptome sequencing

Prior to the library preparation, the concentration of the samples chosen for the sequencing analysis was measured again for better precision of input amounts of RNA using Qubit™ 2.0 (Invitrogen, Thermo Fisher Scientific, Waltham, MA, USA) fluorometer. The integrity of RNA was determined using Agilent 2200 TapeStation system and RNA ScreenTape (Agilent, Santa Clara, CA, USA). As some samples still contained some residual genomic DNA which would interfere with the sequencing of the desired fragments, we removed it using the DNA-free™ DNA Removal Kit (Invitrogen, Thermo Fisher Scientific, Waltham, MA, USA) according to the manufacturer's protocol. The concentration of the pure RNA was measured again using Qubit™ 2.0 (Invitrogen, Thermo Fisher Scientific, Waltham, MA, USA) fluorometer and then we proceeded to the ribosomal RNA depletion. First, the samples were diluted in 26 ul of nuclease-free water (Qiagen, Hilden, Germany) to achieve 500 ng of total RNA input. For the RNA depletion, we used the RiboCop rRNA Depletion Kit V1.2 (Lexogen, Vienna, Austria) according to the manufacturer's protocol in order to eliminate the ribosomal RNA which would overwhelm the sequencing capacity. The concentration of the purified RNA was measured again using Qubit™ 2.0 (Invitrogen, Thermo Fisher Scientific, Waltham, MA, USA) fluorometer.

We then proceeded to the sequencing library preparation using Ultra™ II Directional RNA Library Prep Kit for Illumina® (New England Biolabs, Ipswich, MA, USA), AM-Pure® XP Beads (Beckman Coulter, Brea, CA, USA), and NEBNext® Multiplex Oligos for Illumina® (Dual Index Primers Set 1) (New England Biolabs, Ipswich, MA, USA). Minor adjustments have been made to the manufacturer's protocol: RNA has been fragmented for a longer time (8 min despite higher RNA quality); incubation with the USER Enzyme has been carried out as a first step in the PCR Enrichment of Adaptor Ligated DNA reaction; this reaction has been also run in the Biometra Optical Thermocycler® (Analytik Jena, Jena, Germany) which allows following the amplification curve real-time and stopping the reaction when the desired amplification signal is achieved. To visualize the amplification, we added 2 ul of EvaGreen® Dye, 20X in Water (Biotium, Fremont, CA, USA) into the PCR reaction mix, and individual tubes were taken out of the machine when the fluorescence reached 5000. Purifying beads volume had to be adjusted as the volume of the mixture was lower before the PCR and higher after the PCR. Final DNA libraries were stored at − 20 °C until further processing.

Quality and quantity of the libraries was measured using the Qubit™ 2.0 (Invitrogen, Thermo Fisher Scientific, Waltham, MA, USA) fluorometer and Agilent 2200 TapeStation system and High Sensitivity D1000 ScreenTape (Agilent, Santa Clara, CA, USA) according to the manufacturer's protocol. All 96 samples have been divided into 8 groups, so 12 patients have been sequenced in each run. Also, we stratified samples semi-randomly, as to have tumor and non-tumor tissue and early as well as late-relapse patients in each run to minimize batch effect. Samples have been pooled equimolar at the 4 nM concentration, which has been rechecked using the Qubit™ 2.0 (Invitrogen, Thermo Fisher Scientific, Waltham, MA, USA) fluorometer. The size of each pool was checked using Agilent 2200 TapeStation system and High Sensitivity D1000 ScreenTape (Agilent, Santa Clara, CA, USA) according to the manufacturer's protocol. Pools containing fragments of undesirable length have been purified using Agencourt AMPure XP magnetic beads (Beckman Coulter, Brea, CA, USA).

Following the Illumina Denature and Dilute protocol, the polls have been denatured and diluted to 1,8 pM final concentration and loaded onto a sequencing cassette from NextSeq 500/550 High Output v2 kit, 75 cycles (Illumina, San Diego, CA, USA) and run according to the manufacturer's protocol.

### Data analysis

Raw data from the Illumina NextSeq 500 was converted to fastq using bcl2fastq2 Con-version software (version 2.20.0), and read quality was checked using FastQC (version 0.11.9) (Andrews 2010). Adapter sequences were identified using the Kraken system (version 16-098) (Davis et al. 2013), and poor read ends were removed using Trimmomatic (version 0.39). The 3 'ends with a threshold value less than five and reads shorter than 35 bp have been considered poor and were removed. The modified libraries were mapped with the STAR tool (version 2.7.0d) (Dobin et al. 2013) to the human genome, the sequence of which was downloaded from the GENCODE (version 37) database. During mapping, each reading was allowed to map to up to 20 different locations. Genes were quantified using RSEM software (version 1.3.1) (Li and Dewey 2011), and differentially expressed lncRNAs were identified using the DESeq2 tool (version 1.18.1) (Love et al. 2014) and edgeR package (version 3.30.3). Six samples have failed library preparation resulting in almost no reads, and thus were removed from any subsequent analysis. Another 24 samples were removed due to low number of gene-aligned reads (less than 10 million). Further, 14 samples were removed due to failed DESeq2 normalization and 4 samples were removed due to more issues. Remaining 56 samples were analyzed and the results are shown at the Results section.

### PCR validation of the results

Based on the results of sequencing, 20 candidate lncRNAs were selected. Ten candidates were chosen based on the comparison of early and late-relapse patients (prognostic lncRNAs), other ten were chosen based on the comparison of tumor and non-tumor tissue (diagnostic lncRNAs). In diagnostic lncRNAs, we simply took first ten lncRNAs with assays available in our supplier, Thermo Fisher. In prognostic lncRNAs, we took lncRNAs with base mean > 20 (except TSPEAR due to its low padj value we decided to test it as well). When choosing specific assays from our supplier, we decided to take only assays which spanned exons, had best coverage and possibly the highest RefSeq number.

For the validation of the sequencing results, we used the High-Capacity cDNA Reverse Transcription Kit (Applied Biosystems, Thermo Fisher Scientific, Waltham, MA, USA) for reverse transcription. TaqMan™ Gene Expression Master Mix (Applied Biosystems, Thermo Fisher Scientific, Waltham, MA, USA) was used in qPCR run on QuantStudio 12 K Flex Real-Time PCR System (Applied Biosystems, Thermo Fisher Scientific, Waltham, MA, USA) according to the manufacturer's protocol. TaqMan® Gene Expression Assays used for qPCR along with the catalogue number of product (Assay ID) (Applied Biosystems, Thermo Fisher Scientific, Waltham, MA, USA) are listed in Table 2 below. The expression of PPIA was used as a normalization standard and reference gene based on literature search and our previous experience with the expression of PPIA in RCC tissue (Bohosova et al. 2022).

**Table 2:** Gene Expression Assays used for qPCR along with the catalogue number of product (Assay ID) (Applied Biosystems, Thermo Fisher Scientific, Waltham, MA, USA)

Long non-coding RNA	Assay ID
COLCA1	Hs04186919_m1
ZMIZ1-AS1	Hs00404110_m1
LINC00865	Hs00297721_m1
SNHG3	Hs00903193_m1
LINC00680	Hs03006724_m1
LINC01285	Hs01388470_m1
TSPEAR-AS2	Hs00738388_g1
LINC00472	Hs00227572_m1
ZNF667-AS	Hs00383625_m1
RMST	Hs00327058_m1
PVT1	Hs00413039_m1
CDKN2B-AS1	Hs03300540_m1
LINC01020	Hs03678151_m1
LINC00887	Hs03665538_m1
TCL6	Hs00220956_m1
AZGP1P1	Hs01591157_m1
LINC00462	Hs04332669_g1
LINC00955	Hs01650751_m1
COL18A1-AS1	Hs01651334_m1
LUCAT1	Hs04978593_m1

### Statistical analysis

For the statistical analysis of the validation phase results, we used normalized expression data which were evaluated using Mann–Whitney *U* Test, Wilcoxon test, ROC analysis, and Kaplan–Meier analysis (GraphPad Prism 8, GraphPad Software, La Jolla, CA, USA). P values lower than 0,05 were considered statistically significant. Combined ROC curves were prepared using both GraphPad Prism 8 and JMP software (SAS-JMP software, SAS Institute, Cary, North Carolina, USA). All statistically significant lncRNAs in stage and relapse comparison and all diagnostic lncRNAs with AUC > 0.9200 were analyzed in different combinations of the lncRNAs in the panels. Based on the results of combined ROC analysis for several lncRNAs, a diagnostic Dx score has been developed:

$${\text{DxScore }} = {\text{ Intercept }} - \, \left( {{\text{estimate}}_{{{\text{lncRNA1}}}} *{\text{lncRNA1}}} \right) \, - \ldots . - \left( {{\text{estimate}}_{{{\text{lncRNAx}}}} *{\text{lncRNAx}}} \right)$$

## Results

### lncRNA expression profiles in RCC patients

**Fig. 1:**
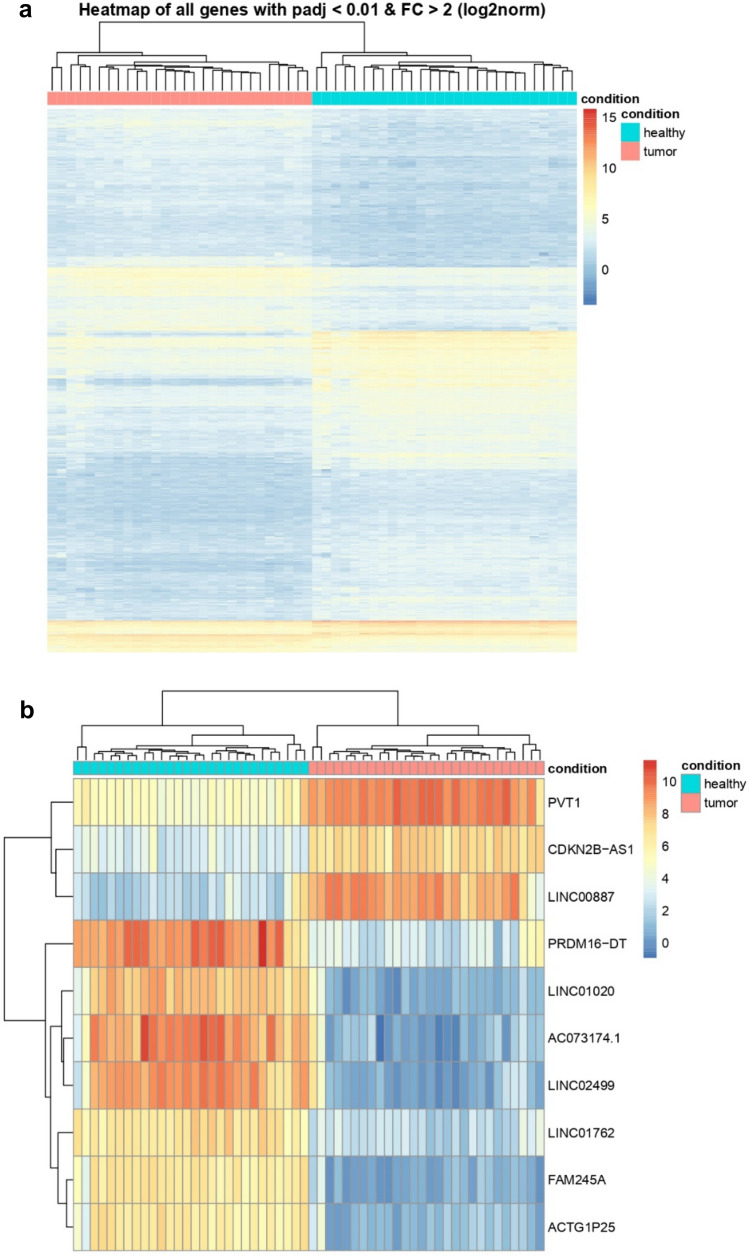
**a** Clastrogram and the heatmap showing 2800 significantly dysregulated lncRNAs in 28 samples of tumor tissue (pink) and s28 non-tumor tissue samples (blue). In the heatmap, red shows higher expression, and blue shows lower expression. lncRNAs with foldchange > 2 and *p* < 0.05 were considered significant. **b** Clastrogram and the heatmap showing showing 20 most significantly dysregulated lncRNAs in 28 samples of tumor tissue (pink) and 28 non-tumor tissue samples (blue). In the heatmap, red shows higher expression, and blue shows lower expression. lncRNAs with foldchange > 2 and *p* < 0.05 were considered significant

Next-generation sequencing results were analyzed using the DESeq2 tool. Comparing the tumor and paired non-tumor tissue, we identified 2800 dysregulated lncRNAs (Fig. 1a, b). Twenty most dysregulated lncRNAs are listed in Table 3. However, when comparing early and late responders, we found only three significantly dysregulated lncRNAs in patients with early relapse of the disease (Table 4). In Tables 5 and 6 are listed all the lncRNAs chosen for validation along with their specific performance in sequencing.

**Table 3:** Twenty most significantly dysregulated lncRNAs in tumor and non-tumor tissue of RCC patients according to the *p* value and adjusted *p* value; *FC* Fold Change in relation to the non-tumor tissue

Gene ID	Gene name	FC	BaseMean	P value	Adjusted p	Gene biotype
ENSG00000285958.1	AC073174.1	– 8.95	423.44	3.93E-71	4.49E-67	lncRNA
ENSG00000249859.12	PVT1	4.14	395.80	8.97E-71	5.12E-67	lncRNA
ENSG00000240498.9	CDKN2B-AS1	4.07	93.45	6.41E-69	2.44E-65	lncRNA
ENSG00000215231.8	LINC01020	– 7.50	129.94	3.09E-64	8.8E-61	lncRNA
ENSG00000250436.1	LINC02499	– 8.99	264.76	3.4E-62	7.76E-59	lncRNA
ENSG00000177133.11	PRDM16-DT	– 5.99	366.09	1.97E-60	3.74E-57	lncRNA
ENSG00000228055.3	FAM245A	– 6.56	49.16	1.86E-58	3.03E-55	lncRNA
ENSG00000214145.7	LINC00887	6.28	300.96	4.71E-57	6.72E-54	lncRNA
ENSG00000233154.6	LINC01762	– 4.31	61.37	2.92E-54	3.7E-51	lncRNA
ENSG00000234996.4	ACTG1P25	– 5.37	48.71	4.59E-54	5.24E-51	transcribed_processed_pseudogene
ENSG00000187621.15	TCL6	– 5.41	547.39	1.61E-51	1.67E-48	lncRNA
ENSG00000214313.8	AZGP1P1	– 5.09	33.93	1.12E-50	1.06E-47	transcribed_unprocessed_pseudogene
ENSG00000275897.1	AC021491.4	– 6.72	74.72	4.61E-50	4.05E-47	lncRNA
ENSG00000233610.1	LINC00462	8.31	71.08	6.08E-50	4.96E-47	lncRNA
ENSG00000214796.8	TUBA5P	– 4.26	90.50	7.27E-50	5.53E-47	transcribed_unprocessed_pseudogene
ENSG00000257027.1	AC010186.3	1.84	213.41	6.83E-49	4.87E-46	lncRNA
ENSG00000216560.4	LINC00955	– 6.46	46.67	3.29E-48	2.21E-45	lncRNA
ENSG00000285783.1	AC098588.3	– 4.84	28.15	5.87E-48	3.73E-45	lncRNA
ENSG00000285662.2	FAM245B	– 5.95	66.96	1.38E-47	8.3E-45	lncRNA
ENSG00000183535.9	COL18A1-AS1	– 5.06	55.01	5.33E-47	3.04E-44	lncRNA

**Table 4:** Twenty most significantly dysregulated lncRNAs in early and late-relapse RCC patients according to the *p* value and adjusted *p* value; *FC* fold change in relation to the late-relapse tissue

Gene ID	Gene name	FC	BaseMean	*P* value	Adjusted p	Gene biotype
ENSG00000196167.10	COLCA1	– 2.70	252.05	6.81E-07	0.009.798	lncRNA
ENSG00000278041.1	AL133325.3	2.80	2.60	1.17E-06	0.009.798	lncRNA
ENSG00000259671.1	MTCYBP23	3.80	3.07	4.35E-06	0.024.214	processed_pseudogene
ENSG00000182912.6	TSPEAR-AS2	3.06	3.26	1.43E-05	0.059.725	lncRNA
ENSG00000232334.1	AL683842.1	1.75	9.67	3.96E-05	0.123.207	processed_pseudogene
ENSG00000226520.1	KIRREL1-IT1	1.25	6.20	4.43E-05	0.123.207	lncRNA
ENSG00000262528.2	AL022341.2	1.57	8.70	6.31E-05	0.135.576	lncRNA
ENSG00000189229.11	AC069277.1	3.21	9.75	6.5E-05	0.135.576	lncRNA
ENSG00000224596.8	ZMIZ1-AS1	1.70	26.96	7.87E-05	0.145.834	lncRNA
ENSG00000224397.7	PELATON	1.63	35.16	0.000.119	0.184.936	lncRNA
ENSG00000280604.1	AJ239328.1	2.46	6.77	0.000.131	0.184.936	lncRNA
ENSG00000237268.2	AC092447.7	2.90	5.36	0.000.133	0.184.936	Transcribed_unprocessed_pseudogene
ENSG00000257512.1	AC124947.2	3.09	2.41	0.000.161	0.20.345	Transcribed_processed_pseudogene
ENSG00000267123.7	SCAT1	1.74	7.04	0.000.182	0.20.345	lncRNA
ENSG00000275613.2	AC243830.1	2.08	5.20	0.000.183	0.20.345	lncRNA
ENSG00000260658.6	AC138305.1	– 2.41	62.07	0.000.196	0.20.439	lncRNA
ENSG00000237463.6	LRRC52-AS1	– 3.78	5.22	0.000.217	0.213.252	lncRNA
ENSG00000261502.4	AC040174.1	–2.19	82.11	0.000.252	0.233.048	lncRNA
ENSG00000229613.2	LINC01501	2.65	3.50	0.000.265	0.233.048	lncRNA
ENSG00000224769.1	MUC20P1	– 2.27	120.04	0.000.314	0.239.852	Unprocessed_pseudogene

**Table 5:** List of candidate lncRNAs chosen for validation based on the comparison of tumor and non-tumor RCC tissue (diagnostic lncRNAs)

Position	Gene ID	Gene name	FC	BaseMean	*P* value	Adjusted p	Gene biotype
2	ENSG00000249859.12	PVT1	4.14	395.80	8.97E-71	5.12E-67	lncRNA
3	ENSG00000240498.9	CDKN2B-AS1	4.07	93.45	6.41E-69	2.44E-65	lncRNA
4	ENSG00000215231.8	LINC01020	– 7.50	129.94	3.09E-64	8.8E-61	lncRNA
8	ENSG00000214145.7	LINC00887	6.28	300.96	4.71E-57	6.72E-54	lncRNA
11	ENSG00000187621.15	TCL6	– 5.41	547.39	1.61E-51	1.67E-48	lncRNA
12	ENSG00000214313.8	AZGP1P1	– 5.09	33.93	1.12E-50	1.06E-47	Transcribed_unprocessed_pseudogene
14	ENSG00000233610.1	LINC00462	8.31	71.08	6.08E-50	4.96E-47	lncRNA
17	ENSG00000216560.4	LINC00955	– 6.46	46.67	3.29E-48	2.21E-45	lncRNA
20	ENSG00000183535.9	COL18A1-AS1	– 5.06	55.01	5.33E-47	3.04E-44	lncRNA
26	ENSG00000248323.7	LUCAT1	4.71	296.56	9.72E-46	4.26E-43	lncRNA

**Table 6:** List of candidate lncRNAs chosen for validation based on the comparison of early- and late-relapsing RCC patients (prognostic lncRNAs)

Position	Gene ID	Gene name	FC	BaseMean	P value	Adjusted p	Gene biotype
1	ENSG00000196167.10	COLCA1	– 2.70	252.05	6.81E-07	0.009.798	lncRNA
4	ENSG00000182912.6	TSPEAR-AS2	3.06	3.26	1.43E-05	0.059.725	lncRNA
9	ENSG00000224596.8	ZMIZ1-AS1	1.70	26.96	7.87E-05	0.145.834	lncRNA
23	ENSG00000232229.6	LINC00865	– 1.83	116.11	0.000.335	0.239.852	lncRNA
24	ENSG00000242125.3	SNHG3	0.84	459.63	0.000.366	0.239.852	lncRNA
38	ENSG00000215190.9	LINC00680	– 0.64	116.39	0.000.629	0.270.476	Transcribed_unprocessed_pseudogene
44	ENSG00000203650.9	LINC01285	1.02	22.91	0.000.828	0.31.403	lncRNA
62	ENSG00000233237.8	LINC00472	– 0.66	3990.65	0.001.874	0.499.278	lncRNA
70	ENSG00000166770.11	ZNF667-AS1	– 1.21	163.03	0.002.098	0.499.278	lncRNA
78	ENSG00000255794.9	RMST	– 1.72	354.20	0.002.401	0.499.278	lncRNA

### qPCR validation of sequencing results

**Fig. 2:**
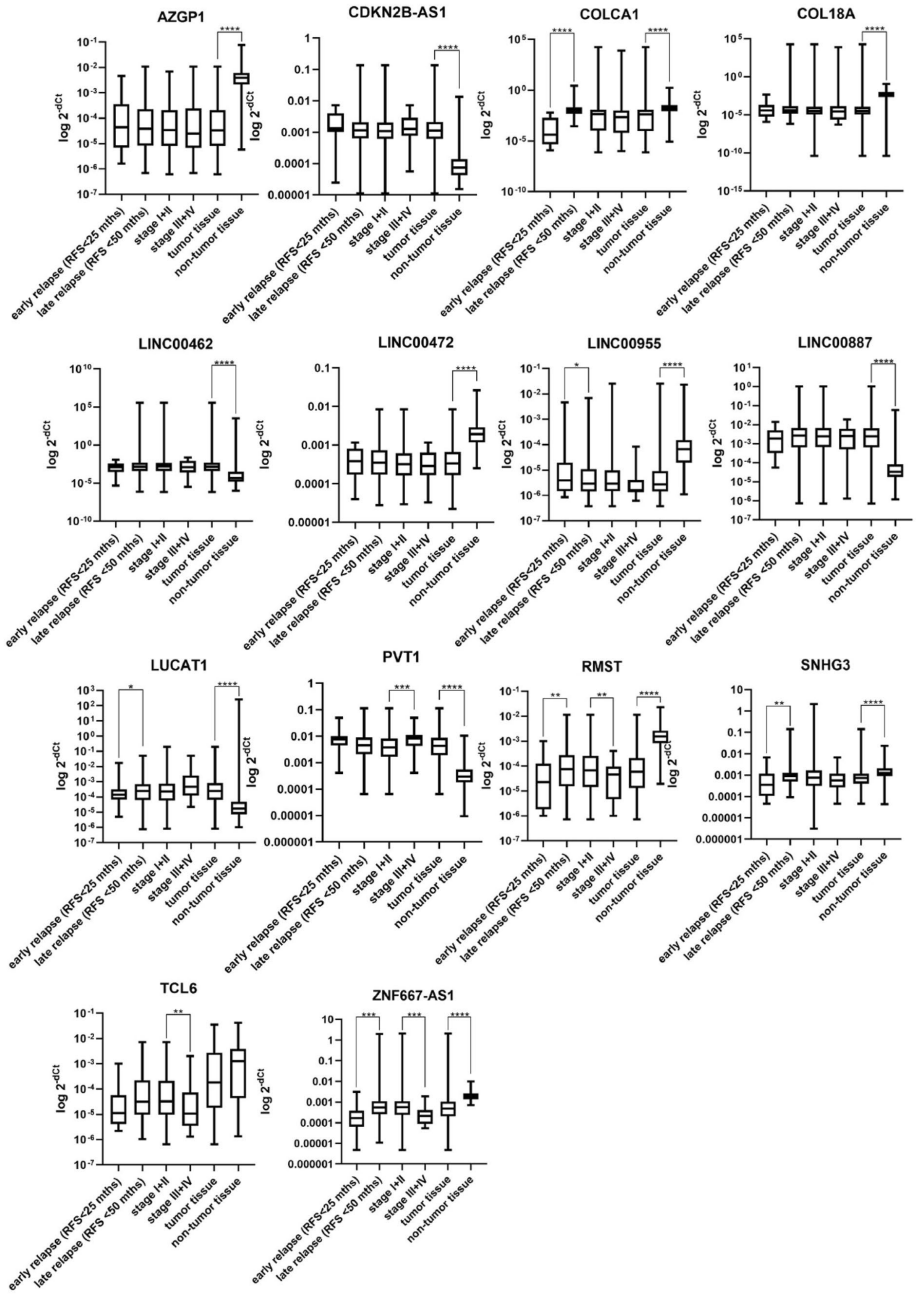
Significantly dysregulated lncRNAs, each graph shows all comparisons analyzed for each lncRNA (tumor vs. non-tumor, stage I + II vs. stage III + IV, early relapse vs. late relapse)

**Fig. 3:**
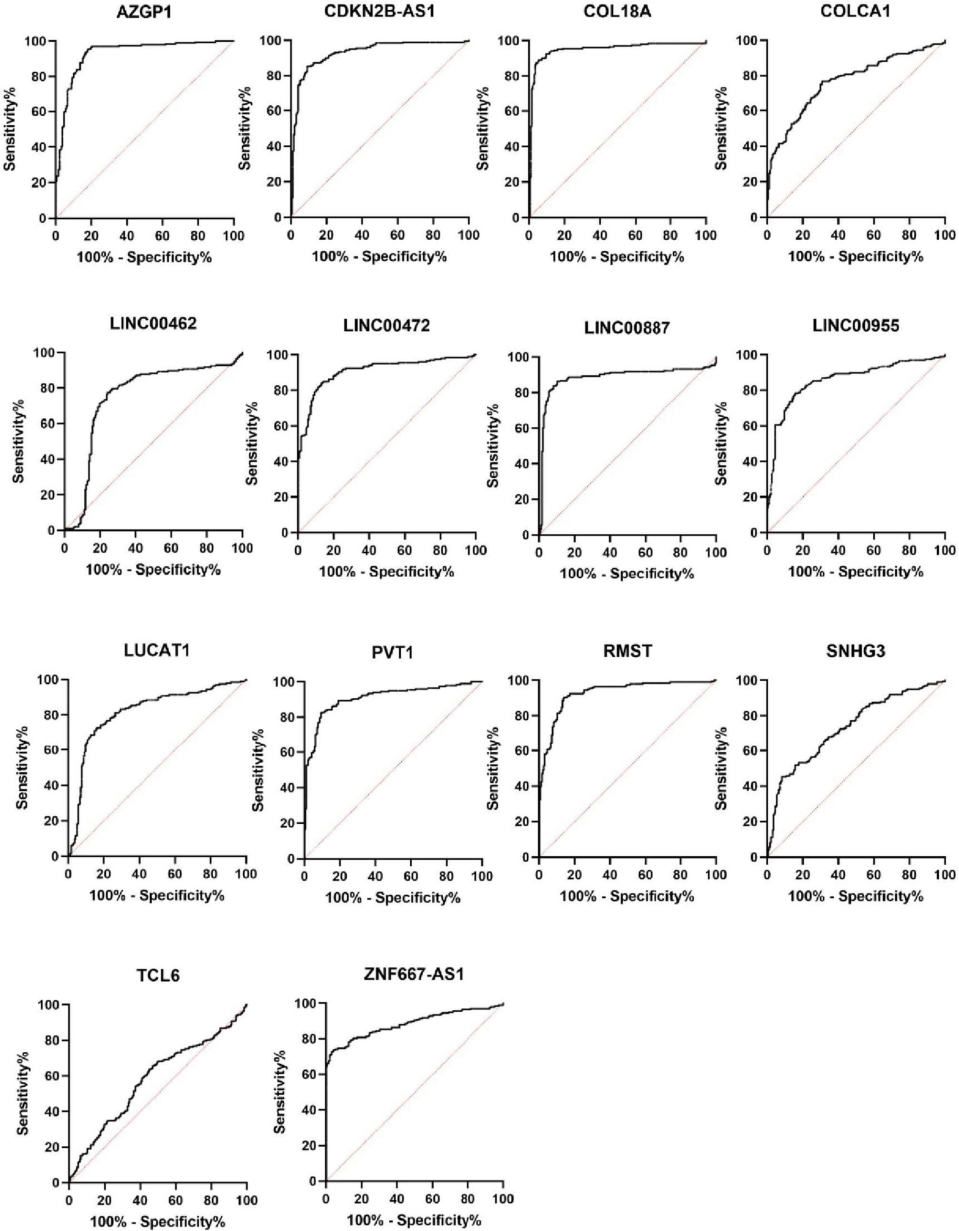
Graph showing ROC curves of candidate lncRNAs dysregulated in tumor tissue compared to non-tumor tissue of RCC patients

**Fig. 4:**
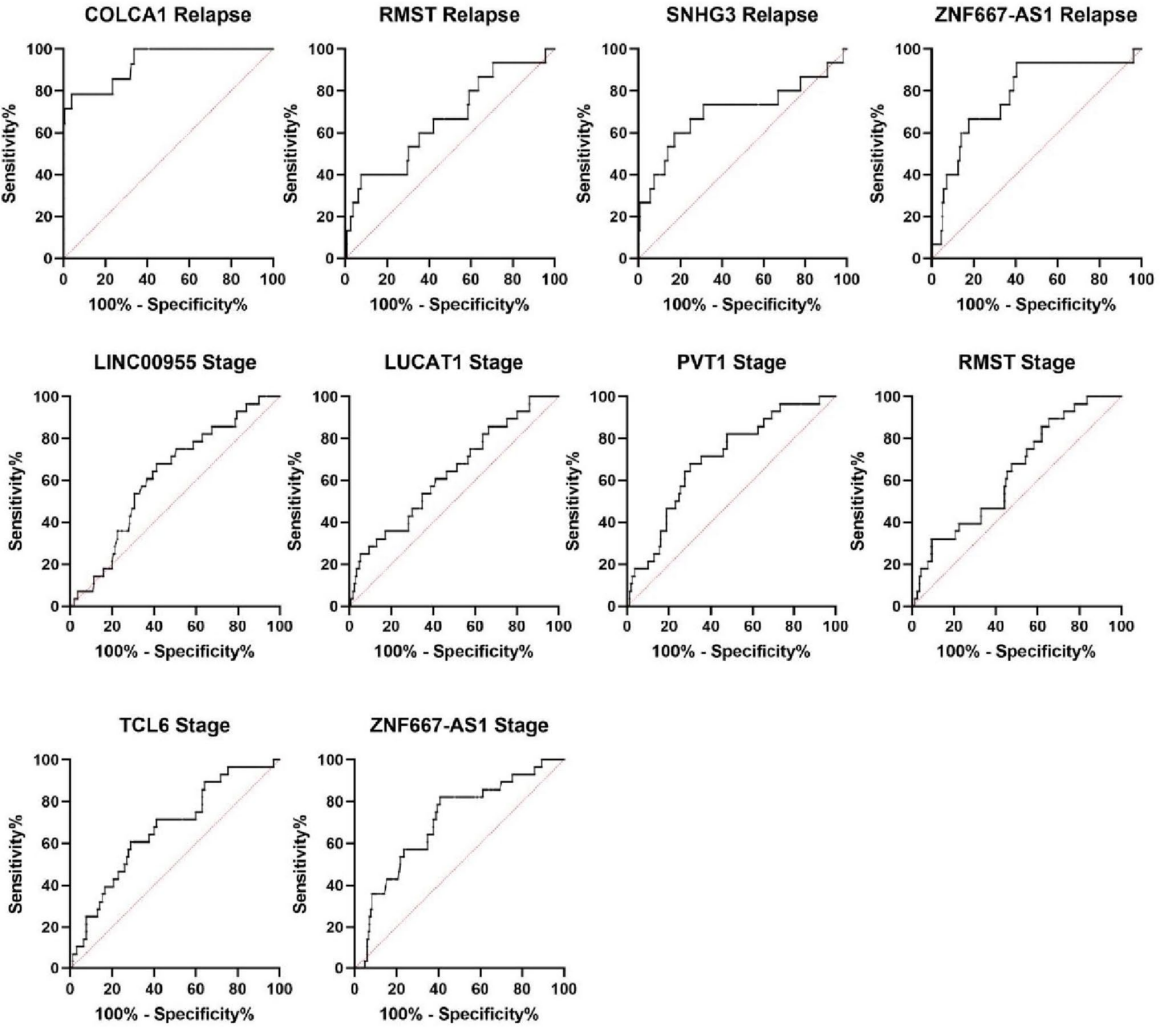
Graph showing ROC curves of candidate lncRNAs dysregulated in tumor tissue of patients with or without early relapse (labeled Relapse) of with different stage of the disease (labeled Stage)

Twenty selected candidate lncRNAs were analyzed using qPCR. Their expression levels have been measured in tumor and paired non-tumor tissue of all patients in the validation cohort and Cq values were normalized to the expression of PPIA. Fourteen lncRNAs have been successfully validated as significantly dysregulated in tumor tissue compared to the paired non-tumor tissue. Four lncRNAs (ZNF667-AS1, RMST, COLCA1, and SNHG3) were significantly dysregulated also in patients with early relapse compared to the late-relapsing patients. We also compared patients in lower stages (stage 1 and 2) with patients in higher stages (stage 3 and 4). Six lncRNAs also shown association with stage of the patients. Results of all lncRNAs with statistically significant dysregulation of expression (in any comparison) are shown in Fig. 2. In all validated lncRNAs we also did ROC analysis (Figs. 3 and 4). Understandably, lncRNAs differed in the ability to distinguish a tumor and non-tumor tissue, however, ten of them showed satisfactory AUC above 0.75. All results are summarized in Table 7.

**Table 7:** Significantly dysregulated lncRNAs and their statistical performance in different comparisons

lncRNA	T v N (*p* value), change in expression, AUC	E v L (*p* value), change in expression, AUC	Stage (*p* value), change in expression, AUC
AZGP1P1	< 0,0001, D, 0,9298	–	–
CDKN2B-AS1	< 0,0001, U, 0,9293	–	–
COL18A1	< 0,0001, D, 0,9548	–	–
COLCA1	< 0,0001, D, 0,7681	< 0,0001, D, 0,9333	–
LINC00462	< 0,0001, U, 0,7519	–	–
LINC00472	< 0,0001, D, 0,9088	–	–
LINC00887	< 0,0001, D, 0,8868	–	-–
LINC00955	< 0,0001, U, 0,8585	–	0,0429, D, 0,6193
LUCAT1	< 0,0001, U, 0,8173	–	0,0241, U, 0,6327
PVT1	< 0,0001, U, 0,9089	–	0,0005, U, 0,7021
RMST	< 0,0001, D, 0,9278	0,0366, D, 0,6629	0,0206, D, 0,6361
SNHG3	< 0,0001, D, 0,7332	0,009, D, 0,7071	–
TCL6	–	–	0,0035, D, 0,6706
ZNF667-AS1	< 0,0001, D, 0,8843	0,0002, D, 0,7803	0,0004, D, 0,7039

*AUC* area under the curve, *D *downregulated, E v L early versus late relapse, T v N tumor versus non-tumor tissue, *U* up-regulated

**Fig. 5:**
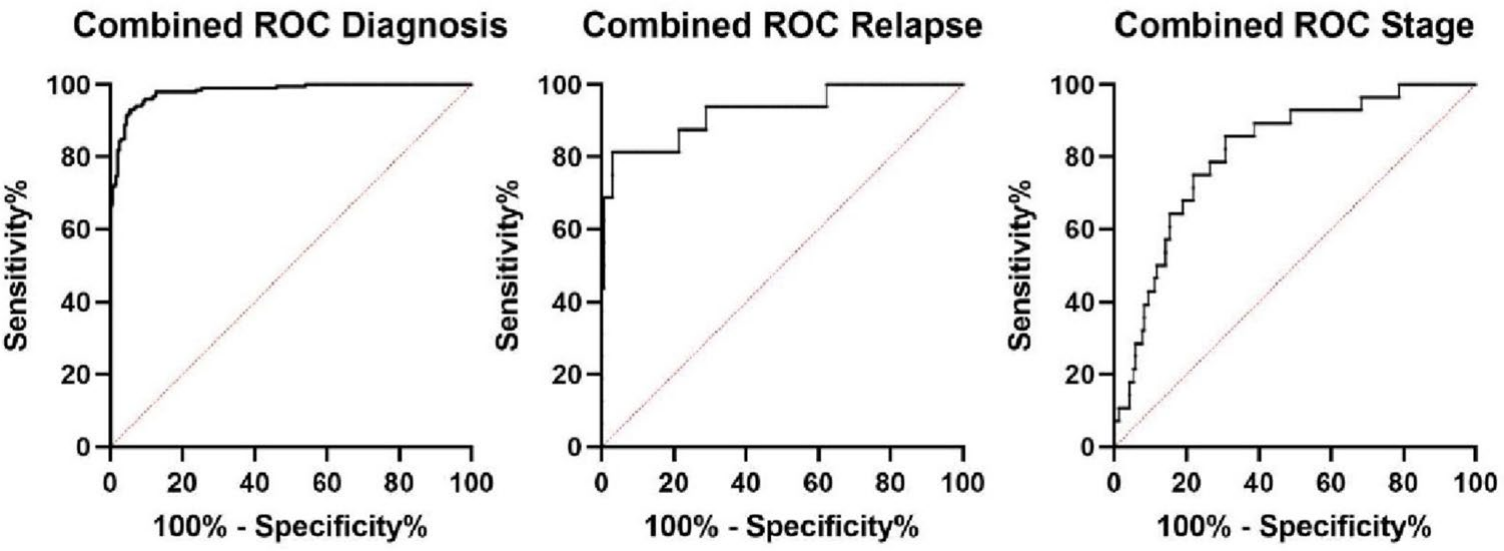
Graph showing combined ROC curves of the most significantly dysregulated lncRNAs diagnostic of prognostic setting

In order to gain better discriminatory results, we combined statistically significantly dysregulated lncRNAs into a diagnostic, and prognostic models. Logistic regression analysis showed that linear combination of AZGP1P1, CDKN2B-AS1, COL18A1, and RMST provided best diagnostic discrimination (AUC 0.9808, sensitivity 95.96%, specificity 90.4%) outperforming other combinations of lncRNAs (Table 8). In stage comparison, the combination of ZNF667-AS1, PVT1, RMST, LINC00955, and TCL6 yielded best results with AUC 0,812, sensitivity 85.71% and specificity 69,41%. And although even if the combination of COLCA1, RMST, SNHG3, and ZNF667-AS1 in relapse comparison did bring higher AUC than RMST, SNHG3, and ZNF667-AS1 alone, it did not outperformed AUC of COLCA1 alone, which makes this lncRNA a superior prognostic biomarker (Fig. 5).

**Table 8:** Results of combined ROC analysis for lncRNA panels achieving the best diagnostic and prognostic values

Type of model	lncRNAs in the model	Combined AUC	Dx value threshold	Sensitivity% (95% CI)	Specificity% (95% CI)	LR
Diagnosis	AZGP1P1 CDKN2B-AS1 COL18A1 RMST	0,9808	0,7717	95,96 (92,23% to 97,94%)	90,4 (85,50% to 93,77%)	10
Prognosis (Early relapse prediction)	COLCA1 RMST SNHG3 ZNF667-AS1	0,9241	5,888	93,75 (71,67% to 99,68%)	71,07 (63,59% to 77,55%)	3,24
Stage	ZNF667-AS1 PVT1 RMST LINC00955 TCL6	0,812	1,427	85,71 (68% to 94,30%)	69,41 (62,12% to 75,85%)	2,802

*CI* confidence interval, *Dx* diagnostic score, *LR* likelihood ratio

## Discussion

In this study, we provide valuable results showing set of exceptional diagnostic and prognostic biomarkers feasible in RCC therapy. Our main aim was to sequence transcriptome of a large set of patients and provide lncRNA expression profiles of a novel and unique patient cohort. We identified more than 2800 significantly dysregulated lncRNAs, out of which we chose twenty for further validation on an independent cohort. Moreover, we aimed to validate diagnostic and prognostic value of selected candidate lncRNAs and their association with early relapse and/or stage, which was successful as 14 out of 20 lncRNAs did perform statistically significantly (*p* < 0.0001 in diagnostic setting, *p* < 0.05 in prognostic setting). To achieve better discriminatory results, we tested several combinations of lncRNAs and identified panels with the best diagnostic and prognostic value. However, in case of early relapse prediction, even the combination achieving the highest AUC did not outperform lncRNA COLCA1. To our best knowledge, we are the first to show that this lncRNA holds such a prognostic power and is significant not only regarding the early relapse after nephrectomy but in RCC in general, as there is only one other work mentioning COLCA1 in relation to RCC as one of nine redox-related lncRNA associated with overall survival of RCC patients (Qi-Dong et al. 2020).

There are however, several other lncRNAs in our panel, which are not frequent in similar RCC-focused studies. For example AZGP1P1 has been associated only with prognosis in breast cancer (Liu et al. 2019), LINC00462, which has been associated with ferroptosis according to one recent work (Wu et al. 2022b), LINC00955 without any other evidence, RMST, which is known to be involved in the development of other tumors, but not RCC (Chen et al. 2022) and similarly ZNF667-AS1, also known as MORT (Vrba and Futscher 2018).

On the other hand, we also identified several notoriously known lncRNAs typically associated with development of RCC. There is CDKN2B-AS1, known also as ANRIL, is up-regulated in RCC tissue which corresponds with our results (Angenard et al. 2019; Dasgupta et al. 2020; Xie et al. 2021) and there are even some single nucleotide polymorphisms identified which are typically associated with RCC (Li et al. 2014). COL18A1 is downregulated as in our study also in other works (Yang et al. 2018; Angenard et al. 2019; Liu et al. 2022) and its expression seems to be specific for chromophobe RCC (He et al. 2016). Dysregulation of LINC00472 along with PVT1 and LUCAT1 has been described in our previous work (Bohosova et al. 2022). LINC00472 has been mentioned also in other works as downregulated in RCC and associated with progression of the disease. Even some mechanisms of LINC00472 functioning have been elucidated (Gao and Wang 2021; Xiang et al. 2021; Wang et al. 2022). PVT1 and LUCAT1 are very well-described lncRNAs in RCC as their impact on overall survival has been shown among several other works also in meta-analysis from Wang et al. (Wang et al. 2019). Involvement of PVT1 into the development of RCC has been thoroughly reviewed also in Bohosova et al. (2021). Similarly, TCL6 has also been shown to be involved in development of RCC as a tumor suppressor (Rysz et al. 2022) and interestingly, artificial increase of TCL6 in cancer cells sensitizes them to paclitaxel, which could pose a novel therapeutical opportunity (Chen et al. 2020).

Although most results correspond with other works, there were some discrepancies in the direction of change in expression, namely in SNHG3 and LINC00887. While SNHG3, an autophagy-related lncRNA (Xuan et al. 2021), was downregulated in our study, other works show its upregulation in RCC (Zhang et al. 2019; Yang et al. 2020; Xu et al. 2021). Similarly, LINC00887 was downregulated in our study and up-regulated in study of Wu et al. (Wu et al. 2022a). However, the main drawback of current state of knowledge regarding the lncRNA dysregulation in RCC lies in the sequencing data, as in majority of works the same set of TCGA sequencing data was analyzed. Therefore, high degree of inter-rater reliability is expected, while some discrepancies between TCGA and our independent cohort are inevitable.

In conclusion, we identified distinct expression profiles of lncRNA in renal tumor tissue and based on our exploratory data, we successfully validated several highly reliable diagnostic and prognostic tissue biomarkers. Moreover, combination of lncRNAs with the best statistical values provided even better discriminatory values. Our study shows that besides diagnosis and overall survival prognostication, lncRNAs can discern also between patients with different length of relapse-free survival and thus should be considered for further independent validation in larger cohorts.

## Data Availability

Raw sequencing data were generated at CEITEC Genomics Core Facility and are publicly available at the Sequence Read Archive under accession number PRJNA929051. Derived data supporting the findings of this study are available from the corresponding author on request.
